# In Silico Design and Characterization of a Multiepitope Vaccine Candidate Against *Brucella canis* Using a Reverse Vaccinology Approach

**DOI:** 10.1155/jimr/6348238

**Published:** 2025-04-15

**Authors:** Vicente Arriagada, Alberto Osorio, Crisleri Carrera-Naipil, Carlos A. Villacis-Aguirre, Cristian Escobar, Nicolás Morales, Danthe Villa, Lien Mardones, Dafne Pérez, Macarena Jara, Raúl E. Molina, Ítalo Ferrari, Sebastián Azocar, Leonardo A. Gómez, Ángel A. Oñate

**Affiliations:** ^1^Laboratory of Molecular Immunology, Department of Microbiology, Faculty of Biological Sciences, University of Concepción, Concepción, Chile; ^2^Simes Educational Center, Santiago, Chile

## Abstract

*Brucella canis* is a Gram-negative bacterium that causes canine brucellosis, a zoonotic disease with serious implications for public health and the global economy. Currently, there is no effective preventive vaccine for *B. canis*. Control measures include diagnostic testing, isolation, and euthanasia of infected animals. However, these measures face significant limitations, such as diagnostic challenges, ethical concerns, and limited success in preventing transmission. Epidemiologically, canine brucellosis exhibits seroprevalence rates ranging from less than 1% to over 15%, with higher rates reported in stray dogs and regions of low socioeconomic development. This study employed a reverse vaccinology approach to design and characterize a multiepitope vaccine candidate against *B. canis*, aiming to prevent infection caused by this pathogen. A comprehensive in silico analysis of the complete *B. canis* proteome was conducted to identify proteins with potential as vaccine targets. Predicted epitopes for B and T cells were analyzed, and those with the highest capacity to elicit a robust immune response were selected. These proteins were classified as plasma membrane proteins, outer membrane proteins (OMPs), or proteins with similarity to virulence factors. Selection criteria emphasized their essential roles in bacterial function, lack of homology with proteins from dogs or mice, and presence of fewer than two transmembrane domains. From this process, four candidate proteins were identified. Epitopes for B and T cells within these proteins were predicted and analyzed, selecting the most immunogenic sequences. The overlap between B- and T-cell epitopes narrowed the selection to six final epitopes. These selected epitopes were then assembled into a multiepitope vaccine construct using flexible linkers to ensure structural integrity and molecular adjuvants to enhance immunogenicity. The physicochemical properties, antigenicity, and toxicity of the designed vaccine were evaluated. Additionally, the secondary and tertiary structure of the vaccine was predicted and refined, followed by a molecular interaction analysis with the Toll-like receptor 4 (TLR4) receptor. The designed vaccine proved to be highly antigenic, nonallergenic, and nontoxic. Validation of its secondary and tertiary structures, along with molecular docking analysis, revealed a high binding affinity to the TLR4 receptor. Molecular dynamics simulations and normal mode analysis further confirmed the vaccine's structural stability and binding capacity. A multiepitope vaccine candidate against *B. canis* was successfully designed and characterized using a reverse vaccinology approach. This vaccine construct is expected to induce robust humoral and cellular immune responses, potentially conferring protective immunity against *B. canis*. The results of this study are promising; however, in vitro and in vivo tests are necessary to validate the vaccine's protective efficacy. Furthermore, the described method could serve as a framework for developing vaccines against other pathogens.

## 1. Introduction

*Brucella* is a monophyletic genus of Gram-negative bacteria comprising at least 13 very similar species. These species exhibit minor genetic differences and display host specificity among several mammals [1]. These bacteria induce brucellosis, a prevalent zoonotic disease transferred among animals or from animals to people via direct contact with blood, urine, placenta or through the inhalation of infected aerosols [2, 3]. It is a priority disease for the World Organization for Animal Health (WOAH) due to its significant impact on public health, the economy, and global trade [4, 5]. This disease is one of the main causes of abortions and infertility in numerous animal species, which leads to great economic losses [6, 7], and human brucellosis remains the most common zoonotic disease worldwide, with more than 500,000 new cases annually [8]. In humans, exposure to brucellosis can cause acute inflammation in several infected organs, displaying irregular fever and flu-like symptoms [9]. The majority of brucellosis cases are caused by the most predominant *Brucella* species in livestock and humans: *B. melitensis*, *B. abortus*, and *B. suis* [10]; however, *B. canis*, the principal etiological agent of canine brucellosis [11] can also be transmitted to humans, causing a neglected and undervalued disease [12]. Canine brucellosis is characterized by a high seroprevalence worldwide in dogs and, consequently, a high likelihood of transmission to humans [13]. This disease is transmitted among dogs through genital contact, oronasal mucosae, and conjunctival tissues [11, 14, 15]. It is considered a venereal infection when *B. canis* is shed in the semen of infected dogs, which can occur during the first weeks after infection or persist for years in these animals [11, 14, 15]. Additionally, infection with *B. canis* is associated with high neonatal mortality rates [16]. However, surviving infected puppies may become significant transmission sources, as they can be permanent carriers of the bacterium [17]. Although the host range of *B. canis* predominantly includes domestic dogs, specific antibodies against *B. canis* antigens have been detected in several mammal species. Experimental infections have also been recorded in goats, cattle, pigs, sheep, and chimpanzees using *B. canis* [18]. In humans, infection with this bacterium tends to produce nonspecific symptoms, similar to other *Brucella* infections, causing intermittent fever, fatigue, and lymphadenopathy [19, 20]. From an epidemiological perspective, *B. canis* seroprevalence rates in dogs range from less than 1% to 15% or higher, with elevated rates observed in stray dogs and impoverished areas. This is likely attributed to a higher proportion of intact dogs and uncontrolled breeding within these populations [13, 21]. Moreover, *B. canis* infections in humans are often underdiagnosed due to their nonspecific symptoms [21]. Recently, a notable increase in canine brucellosis cases has been reported in Great Britain, raising concerns about its potential impact on human health [22]. This increase is alarming because there are currently no specific treatments or effective cures for this disease. Antibiotic therapy has shown limited success in controlling the infection, and when the primary goal is to prevent disease transmission in dogs, euthanasia remains the most reliable measure for both symptomatic and asymptomatic carriers [23]. Given these challenges—such as the close proximity of infected dogs to humans and the lack of effective therapies—there is an urgent need to develop preventive measures. Developing effective canine vaccines capable of eliciting a specific immune response against *B. canis* is a promising approach to controlling the disease in dogs and reducing its transmission to humans [24–26]. Protective immunity against *B. canis* should focus on developing of INF-*γ* secreting type-1 T helper cells (Th1), effectively responding to intracellular pathogens. This type of response must be induced by effective vaccines targeting this bacterium [27, 28]. Despite significant efforts in the field of *Brucella* vaccinology [29], studies specifically aimed at developing vaccines for canine brucellosis are limited and have been confined to the last decade [30, 31]. Notable experimental vaccines against *B. canis* include attenuated vaccines based on mutations in the *vir*B genes that encode the type IV secretion system (T4SS) of *B. canis*, or these based on recombinant proteins using outer membrane proteins (OMPs) such as Omp31 or recombinant VirB proteins [30–33]. Recently, a heterologous vaccine based on *B. ovis* mutant for ABC transporter (Δ*abcBA*) has shown protection against *B. canis* challenge in mice [34]. Progress in genomes, proteomics, and bioinformatics is essential for identifying antigenic peptides from specific diseases, hence aiding in the design and manufacture of next-generation vaccines [35]. Initially, these analyses involved a filtering process of the proteome's total sequences to identify the pathogen's essential proteins, prioritizing those located subcellularly with potential antigenic, nonallergenic, and nontoxic epitopes for B cells and T cells to stimulate a safe and robust humoral and cellular immune response [36]. This approach has recently led to an increase in using bioinformatics tools to develop chimeric vaccines, selecting conserved or superimposed epitopes capable of generating specific B- and T-cell responses (CD4+ and CD8+) [36, 37]. Moreover, linker sequences, which are short amino acid sequences that separate epitopes, play a critical role in multiepitope vaccines by ensuring proper epitope processing and presentation by the host's immune system. This is achieved by preventing the formation of junctional epitopes, which could otherwise compromise the specificity of the immune response. Additionally, linkers contribute to the chimera's flexibility and structural integrity, preserving its overall stability and functionality as a vaccine candidate [38]. Proper placement of linkers can further enhance the immunogenicity of the epitopes, thereby improving the development of a robust and specific immune response [39]. Another crucial component of vaccine design is the use of adjuvants, which enhance the immune response to the target antigen. Classical adjuvants, such as aluminum salts or emulsions (e.g., Freund's adjuvant), function by stimulating the innate immune system and promoting antigen presentation to activate adaptive immunity. However, these adjuvants are often administered separately from the antigen, which may lead to limited effectiveness or side effects [40, 41]. In contrast, *built-in adjuvants* are genetically or chemically incorporated directly into the vaccine construct, allowing for a more targeted and synergistic enhancement of the immune response. These adjuvants can include specific immunostimulatory molecules, such as Toll-like receptor (TLR) agonists, cytokines, or other immune-enhancing domains, which are integrated into multiepitope vaccines to optimize immunogenicity [42, 43]. This approach ensures close spatial and temporal association between the antigenic epitopes and the adjuvant, improving vaccine efficacy while potentially reducing adverse effects.

Therefore, this study focuses on extracting and identifying immunogenic epitopes from the *B. canis* proteome to design a multiepitope canine vaccine candidate. The goal is to effectively stimulate B- and T-cell responses and confer robust protection against this pathogen.

## 2. Materials and Methods

**Figure 1:**
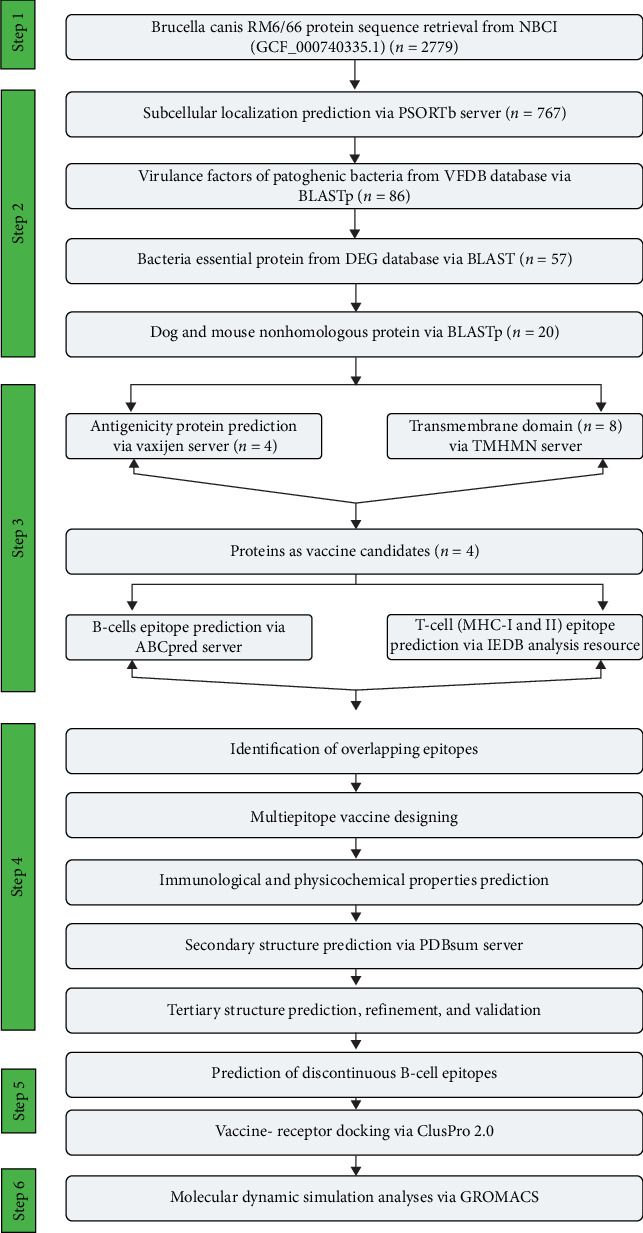
Workflow diagram of the current study illustrating the sequential use of proteomics and reverse vaccinology techniques in designing the multiepitope vaccine for *B. canis*.

Figure 1 illustrates a flow diagram for the design of the multiepitope vaccine candidate against *B. canis*. This candidate's design required the use of a series of servers and programs described sequentially in the text. Table S1 provides a detailed list of the servers and databases used in the vaccine design, specifying their role or function in the study and their availability. Additionally, Table S2 presents the cutoff values applied in the different servers and programs used throughout the analysis.

### 2.1. Search for Candidate Proteins

A representative *Brucella canis* RM6/66 strain genome (GCF_000740335.1) was retrieved from the NCBI GenBank database (https://www.ncbi.nlm.nih.gov/genbank) [44]. This sequence was analyzed using the PSORTb v.3.0.3 (https://www.psort.org/psortb/index.html) server to identify the subcellular localization of proteins, which served as a basis for selecting proteins located in the outer membrane, periplasmic space, plasma membrane, and extracellular regions [45]. The next step involved selecting proteins homologous to known virulence factors as referenced in the Virulence Factors of Bacterial Pathogens Database (VFDB) (http://www.mgc.ac.cn/VFs/main.htm), using BLASTp [46, 47]. Essential proteins were selected, which refer to the genes that an organism needs to survive under certain specific conditions; this search was carried out through the Database of Essential Genes (DEG) (http://origin.tubic.org/deg/public/index.php) [48]. The search also extended to identifying nonhomologous sequences to proteins specific to *Canis familiaris* (dogs) and *Mus musculus* (mice) as found in the NCBI database [49]. Proteins associated with virulence factors, identified as essential and nonhomologous to dogs and mice, were further analyzed for their antigenicity, presence of transmembrane domains, signal peptide, and topology using specialized servers: VaxiJen v2.0 (https://www.ddg-pharmfac.net/vaxijen/VaxiJen/VaxiJen.html), ANTIGENpro (https://scratch.proteomics.ics.uci.edu), TMHMM-2.0 (https://services.healthtech.dtu.dk/services/TMHMM-2.0/), and DeepTMHMM (https://dtu.biolib.com/DeepTMHMM), respectively [50–53].

### 2.2. Prediction of Epitopes for B and T Cells

The proteins identified in the previous steps were used to predict B- and T-cell epitopes. Linear B-cell epitopes were predicted using the ABCPred server (http://crdd.osdd.net/raghava/abcpred/) [54], while CD8^+^ T cells MHC class I and CD4^+^ T cells MHC class II epitopes were identified from the Immune Epitope Database (IEDB) analysis resource (https://www.iedb.org) [55]. The binding of peptides to MHC class I molecules was predicted with the stabilized matrix method (SMM) [56], and for MHC class II molecules, the NetMHCIIpan 4.1 method was used [57]. The mouse H-2 reference allele set with an IC50 of less than 200 nM was applied [58]. Following this, the B- and T-cell epitopes were aligned using MEGA 11 software [59] to identify the overlapping epitopes for B, CD8^+^, and CD4^+^ T cells, which are the focus of further analysis. The antigenicity of these peptides was assessed using ANTIGENpro [52], the allergenicity was predicted using AllerTOP2.0 (https://www.ddg-pharmfac.net/AllerTOP/) [60], the toxicity by ToxinPred2 (https://webs.iiitd.edu.in/raghava/toxinpred2/) [61] and their potential to induce IFN-*γ* was evaluated through IFNepitope servers (http://crdd.osdd.net/raghava/ifnepitope/) [62], respectively.

### 2.3. Design of the Multiepitope Vaccine

To develop the multiepitope chimeric vaccine, the chosen overlapping epitopes were connected using a GSGS flexible linker, with a CTB (a nontoxic component of the cholera toxin's B subunit) was used as an adjuvant sequence at the N-terminal end to boost immunogenicity. Cholera toxin subunit b as adjuvant increases antigenic presentation, B-cell differentiation, as well as cytokine production and their interaction with T cells [63], connected by an EAAAK rigid linker. Additionally, a TpD (an epitope known as a potent universal adjuvant for CD4 T-cell helper [64] sequence was attached at the C-terminal end of the chimeric protein via another GSGS flexible linker; connectors are crucial to prevent the binding among epitopes [65]. Therefore, increased flexibility allows for greater variation in distance between the N and C termini of the fusion protein, impacting antigenic presentation by effectively separating embedded domains of interest [66, 67]. Including lysine (K) in vaccine, constructs enhances solubility by avoiding B-cell epitopes and offers improved immune response while also providing a favorable cleavage site for proteases within lysosomes, an essential step in CD4 T-cell presentation [68, 69].

### 2.4. Antigenicity, Allergenicity, Toxicity, and Physicochemical Parameters

The antigenicity of this chimeric protein was evaluated using VaxiJen v2.0 [50] and ANTIGENpro [52] servers. Allergenicity predictions for the vaccine construct were made using the AllerTOP2.0 server [60], and toxicity assessments were carried out using the ToxinPred2 server [61]. Physicochemical parameters, such as theoretical isoelectric points, molecular weight, and hydrophilicity, were analyzed using the ProtParam server (https://web.expasy.org/protparam/) [70]. Lastly, the SOLpro server (https://scratch.proteomics.ics.uci.edu) was employed to predict the solubility propensity of the vaccine construct [71].

### 2.5. Secondary Structure and Intrinsic Disorder Analyses

To assess the structural integrity of the designed vaccine´s peptide sequence, its secondary structure was determined using the PDBsum server (https://www.ebi.ac.uk/thornton-srv/databases/pdbsum/) [72]. Additionally, intrinsic disorder was performed using IUPred3 (https://iupred3.elte.hu), evaluating regions with a higher propensity for the structural disorder based on the amino acid composition [73].

### 2.6. Tertiary Structure Analysis and Validation

using the AlphaFold Server (https://alphafoldserver.com) [74]. This model was then refined using the GalaxyRefine server (https://galaxy.seoklab.org/cgi-bin/submit.cgi?type=REFINE) [75], which employed soft and aggressive relaxation methods, side chain reconstruction, and molecular dynamics simulation. Postrefinement, the vaccine's 3D structure was validated by ERRAT (https://www.doe-mbi.ucla.edu/errat/), which analyzed the interactions with the environment [76], and by ProSA-web (https://prosa.services.came.sbg.ac.at/prosa.php) for energy analysis [77]. The model´s geometry was evaluated using MolProbity (http://molprobity.biochem.duke.edu) [78], and the stereochemical quality was assessed with the PROCHECK tool (https://www.ebi.ac.uk/thornton-srv/software/PROCHECK/), generating a Ramachandran plot and Wiring Diagram to examine the incorporated residues ‘geometry within the vaccine structure [79].

### 2.7. Prediction of Discontinuous B-Cell Epitopes

Once the 3D protein structure model was refined, the discontinuous B-cell epitopes were predicted using the ElliPro antibody epitope prediction server (http://tools.iedb.org/ellipro/) [80]. This server uses algorithms based upon values of protrusion index (PI) values for measuring the PI of the residues, estimating the shape of the protein as an ellipsoid, and adjacent cluster residues [81]. The vaccine construct was uploaded in PDB format, and all default parameters were kept for epitope prediction.

### 2.8. Structural and Functional Characterization of Vaccine Construct: Engineering Disulfides, Signal Peptide, and Transmembrane Topology

Disulfide engineering introduces disulfide bonds between protein residues. These bonds are covalent interactions that increase the stability of the protein structure by decreasing conformational entropy [82]. Disulfide by Design 2 v2.13 (http://cptweb.cpt.wayne.edu/DbD2/) was used to disulfide engineer the 3D protein structure of the vaccine construct [83]. Residue pairs with an energy value lower than 2.2 kcal/mol and a *χ*^3^ angle between –87° and +97° within this range were chosen for disulfide bond formation, given that 90% of naturally formed disulfide bonds exhibit these characteristics [84]. Additionally, the signal peptide was predicted using the SignalP-6.0 server (https://services.healthtech.dtu.dk/services/SignalP-6.0/), which detects five types of signal peptides through a machine-learning model [85]. The transmembrane topology was predicted using the DeepTMHMM server (https://dtu.biolib.com/DeepTMHMM), which predicts and classifies protein topology [53].

### 2.9. Molecular Docking

Molecular docking was performed using ClusPro 2.0 (https://cluspro.bu.edu/login.php), a fast rigid-body protein–protein docking server, to predict interactions with ligands such as the TLR4 [86]. The crystallized structure of the TLR4/MD-2/lipid IVa complex from *M. musculus* (PDB ID 3VQ1) [87] was retrieved from the RCSB Protein Data Bank (https://www.rcsb.org) [88]. We selected a TLR4 chain and MD-2 chains, which were manually extracted from the PDB file and then uploaded to the ClusPro 2.0 server for subsequent docking analysis. The resulting models were further refined using the CHARMM-GUI (https://www.charmm-gui.org) molecular mechanics package [89].

### 2.10. Normal Mode Analysis

Normal mode analyses were conducted on the free-form vaccine construct to describe the flexible states accessible to a protein about an equilibrium position, and for this purpose, we utilized a local deployment of the WEBnma3 API (https://apps.cbu.uib.no/webnma3) [90] to evaluate inter-residue correlations and to scrutinize potential conformational changes. This API applies elastic network models, offering a reliable and cost-effective computational method to analyze the vaccine construct. A single structure analysis was performed, and the workflow automatically calculated normal modes, average deformation energies, fluctuations and atomic displacement analysis, protein trajectories for specified modes, and correlation analysis, providing insight and validation into the dynamic properties of the vaccine construct.

### 2.11. Molecular Dynamic Simulations (MDSs)

MDSs were performed for the structural analysis of the refined construct both in its free form and complexed with TLR4/MD-2. The following procedures were executed for both types of analysis: (a) Ensemble preparation: The CHARMM-GUI server was used to generate a GROMACS input using its Solution Builder tool, with the TIP3P water model employed to fill the cubic space disposed for each experiment [91, 92]. Monte Carlo methods were applied to add K^+^ and Cl^−^ ions to each system until it reached the neutralization of the system. The system was described using the Amber Force Field ff19sb [93]. (b) Minimization: The system's potential energy was minimized using the steepest descent minimization algorithm. (c) Equilibration: The system was equilibrated using *NVT* and *NpT* ensembles targeted at 300K temperature and 1 bar pressure. (d) Production: The final MDS for each scenario was carried out for 30 ns, with an integration step of 2 fs, and energies were recorded every 10 ps. To measure the conformational stability of the vaccine construct and the vaccine-TLR4 complex forms post 30 ns simulations, root mean square deviation (RMSD) profiles for backbone residues, radius of gyration (Rgyr), and root mean square fluctuation (RMSF) from C-alpha atoms were analyzed.

### 2.12. Binding-Free Energy

The binding-free energies of the docked complex were estimated using the MM-PBSA method [94]. The gmx_MMPBSA tool was employed for these calculations [95]. For the binding energy analysis, 1000 frames were extracted at regular intervals from the simulation trajectories.

### 2.13. Codon Optimization and In Silico Cloning of the Vaccine Construct

Codon optimization is needed to improve the rate of expression of a foreign gene in the host organisms [96]. The tool EMBOSS Backtranseq (https://www.ebi.ac.uk/jdispatcher/st/emboss_backtranseq) was used for reverse translation and codon optimization of the vaccine construct in *E. coli* (strain K12 HIGH) [97]. The results of the codon optimization were assessed using the GenScript [98] Rare Codon Analysis Tool (https://www.genscript.com/tools/rare-codon-analysis/). The in silico cloning of the vaccine construct was performed using SnapGene software (https://www.snapgene.com). The pET-28a (+) expression vector, obtained from the Addgene database (https://www.addgene.org/) [99], was selected as the cloning vector. The codon-optimized vaccine construct sequence was modified to include the Shine–Dalgarno sequence, a stop codon, and restriction sites for EcoRI and BamHI enzymes at the 5′ and 3′ ends, respectively. After cloning, the vector was subjected to a 1% agarose gel electrophoresis simulation. In the simulation, the lanes were loaded with a molecular weight ladder, the vaccine construct, the pET-28a (+) vector, and the result of the cloning process.

## 3. Results

In this study, we employed a reverse vaccinology approach to select vaccine candidates from extracellular, periplasmic, and OMPs of *B. canis*. Immunodominant B- and T-cell epitopes were identified within these proteins, and they were assembled using appropriate amino acid connectors (linkers) and molecular adjuvants to create a novel multiepitope vaccine.

### 3.1. Selection of Vaccine Candidates

**Figure 2:**
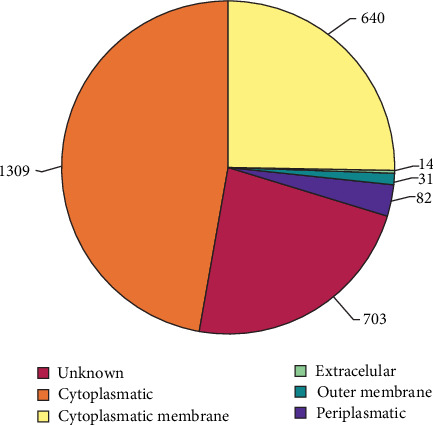
Quantitative representation of the subcellular localization of *B. canis* proteins as predicted by the PSORTb server.

The genome of *B. canis*, obtained from the GCF_000740335.1 database, revealed a total of 2779 proteins identified directly from the genomic data. Analysis using the PSORTb server indicates that 1309 proteins are cytoplasmic, 640 are associated with the cytoplasmic membrane, 14 are extracellular, 31 are OMPs, 82 are periplasmic proteins, and 703 proteins have an undefined cellular location (Figure 2). Proteins classified as cytoplasmic or with undefined cellular localization were excluded for further analyses. Subsequent alignment with the VFDB for the *Brucella* genus using BLASTp revealed that 84 of the 753 extracellular, cytoplasmic membrane and OMPs are known virulence factors. Next, 57 essential proteins were identified by comparing them with the database comprising the essential genes (DEG). To eliminate homologous proteins found in dogs and mice, a BLASTp search against the proteome of these species was conducted, narrowing the candidates to 20 proteins. Finally, out of the 20 selected proteins, only four were chosen as vaccine candidates for possessing fewer than two transmembrane regions, thus avoiding the selection of epitopes located within these domains. These proteins include three cytoplasmic membrane proteins (WP_004684587.1, WP_004690079.1, WP_004692008.1) and one OMP (WP_006133077.1). The scores obtained from PSORTb, VFDB, and DEG servers for these candidate proteins are detailed in Table S3. According to VaxiJen, each candidate exhibited an antigenic potential score ranging from 0.4321 to 0.7680. Additionally, scores from ANTIGENpro ranged from 0.099321 to 0.883782. Furthermore, the molecular weight of these proteins is below 100 kDa, and only one of them (WP_004684587.1) contains both a transmembrane region and a signal peptide. Regarding topology, three of these proteins exhibit a globular topology, while the protein WP_006133077.1 has a beta topology (Table 1).

### 3.2. Prediction of Epitopes for B Cells and T Cells

**Figure 3:**
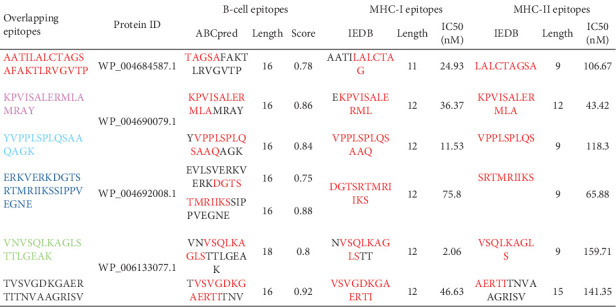
B- and T-cell epitopes predicted using ABCPred and IEDB.

**Figure 4:**
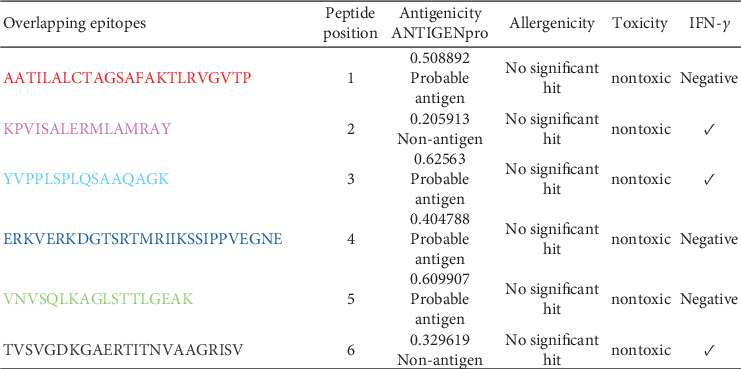
The predicted overlapping epitopes, antigenicity, allergenicity, toxicity, and potential interferon-gamma inducing.

Upon selecting the peptides and aligning the 16-mer sequences for B and the 8–11-mer sequences for T cells with their respective protein sequences, six overlapping epitopes were identified (Figure 3). These six overlapping epitopes were determined to be nonallergenic and nontoxic; furthermore, three were predicted to induce an INF-gamma response (Figure 4). When looking at the antigenicity assessment of the overlapping epitopes, some peptides fell into the nonantigen category (Figure 4), but these were not eliminated due to their high scores observed in the B- and T-cell epitope predictions independently (Figure 3).

### 3.3. Design of the Multiepitope Chimeric Vaccine

**Figure 5:**
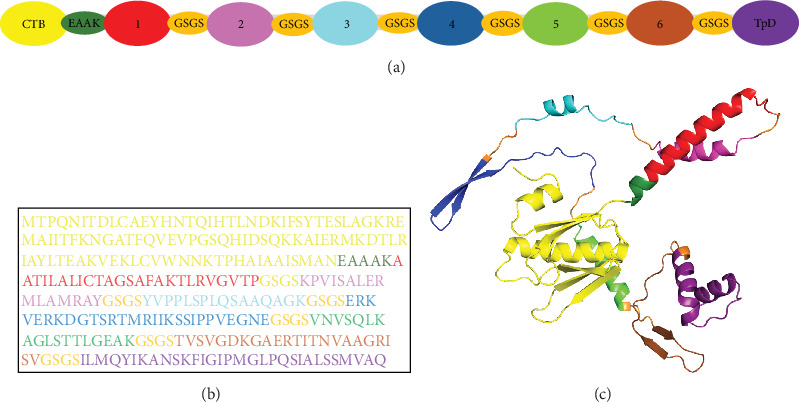
Design and structural modeling of the multiepitope vaccine against *B. canis*. (A) Order of binding for the predicted overlapping antigenic epitopes and their adjacent adjuvant sequences. (B) The sequence of the multiepitope vaccine. (C) Refined 3D structure of the prioritized vaccine construct.

The six peptides were connected with flexible GSGS linkers and flanked with the CTB and TpD adjuvant sequences at the N- and C-terminal ends, respectively. The CTB sequence was connected using the rigid EAAAK linker, while the TpD epitope was linked with GSGS (Figure 5A,B). The AlphaFold server performed the tertiary structure prediction of the vaccine protein (Figure 5C), which was then refined by GalaxyRefine to derive the 3D structural model. This vaccine construct demonstrated high antigenicity, evidenced by VaxiJen and ANTIGENpro scores of 0.74 and 0.93, respectively. SOLpro analysis indicated a solubility probability of 0.52 for the protein. Additionally, the model is considered nonallergenic and nontoxic (Table 2). Physicochemically, the construct has an optimal molecular weight of 30.3 kDa for recombinant production in bacterial expression systems. It has a GRAVY index of −0.117, reflecting its hydrophilic nature. The theoretical isoelectric point (pI) was determined to be 9.66, with an aliphatic index of 84.15 and an instability index of 30.21 (Table 2). These physicochemical properties suggest the vaccine construct is thermostable across various temperature conditions.

### 3.4. Prediction of Secondary Structure

**Figure 6:**
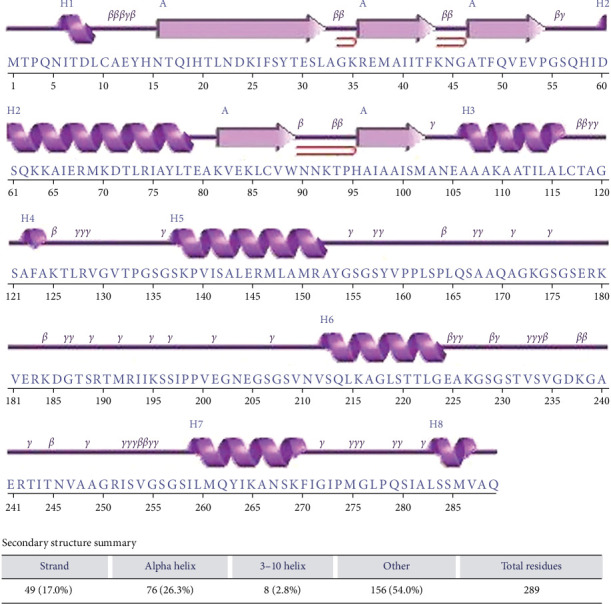
The secondary structure prediction of the vaccine design as performed by the PSIPRED server.

The vaccine construct's secondary structure prediction was composed of 17% *ß*-turns, 26.3% *α*-helices, 2.8% 3–10 helices, and 54% other structures (Figure 6). The intrinsic disorder analysis revealed that the vaccine construct contains regions with a higher degree of disorder, which may suggest potential interaction sites (Figure S1).

### 3.5. Prediction, Refinement, and Validation of Tertiary Structure

**Figure 7:**
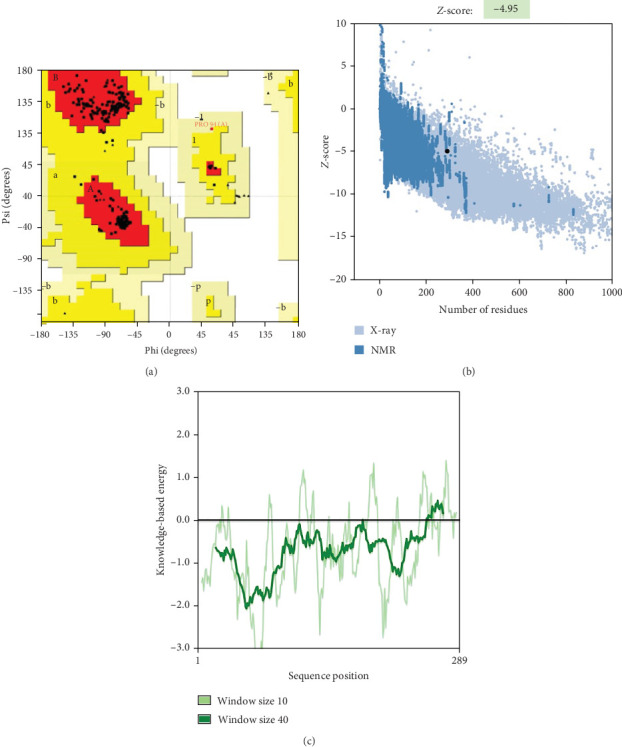
Structural assessment of the multiepitope vaccine construct for *B. canis*. (A) Ramachandran plot: Ramachandran plot statistics display 97.2% of residues within the most favorable regions and 2.8% in allowed regions. (B) Overall model quality from ProSA-web analysis showing a *Z*-score of −4.95 and (C) local model quality for the prioritized vaccine construct.

The AlphaFold server performed the tertiary structure prediction of the vaccine protein (Figure 5C), which was then refined by GalaxyRefine to derive the 3D structural model. The stereochemical quality of the model was assessed with a Ramachandran plot, revealing that 97.2% of amino acid residues are in the most favored regions and 2.8% in allowed regions (thus, 100% are within the favored regions) (Figure 7A). The overall quality factor from ERRAT for the 3D model is 98.0198. The confidence score (*Z* score) for the 3D model is −4.95 according to ProSA web (Figure 7B). These validations suggest that the vaccine construct's tertiary structure is accurate compared to experimentally predicted proteins.

### 3.6. Prediction of Discontinuous B-Cell Epitopes

A total of 11 discontinuous B-cell epitopes were predicted for the vaccine construct. These epitopes had scores between 0.507 and 0.935. In total, 141 amino acids were identified within these 11 epitopes, with sizes ranging from 4 to 35 amino acids (Table 3). These predictions suggest that the vaccine construct could contain several discontinuous epitopes.

### 3.7. Structural and Functional Characterization of the Vaccine Construct

The Disulfide by Design 2 server predicted 18 residue pairs of vaccine construct capable of forming disulfide bonds (Table S4). However, none of these pairs met the energy threshold of less than 2.2 kcal/mol or the *χ*^3^ angle between –87° and +97°. The signal peptide predicted using SignalP indicated that the vaccine construct was classified as “other,” meaning it does not possess a signal peptide (Figure S2). The transmembrane topology prediction was performed using DeepTMHMM, which classified the structure as globular (Figure S3).

### 3.8. Vaccine-TLR4 Docking Analysis

**Figure 8:**
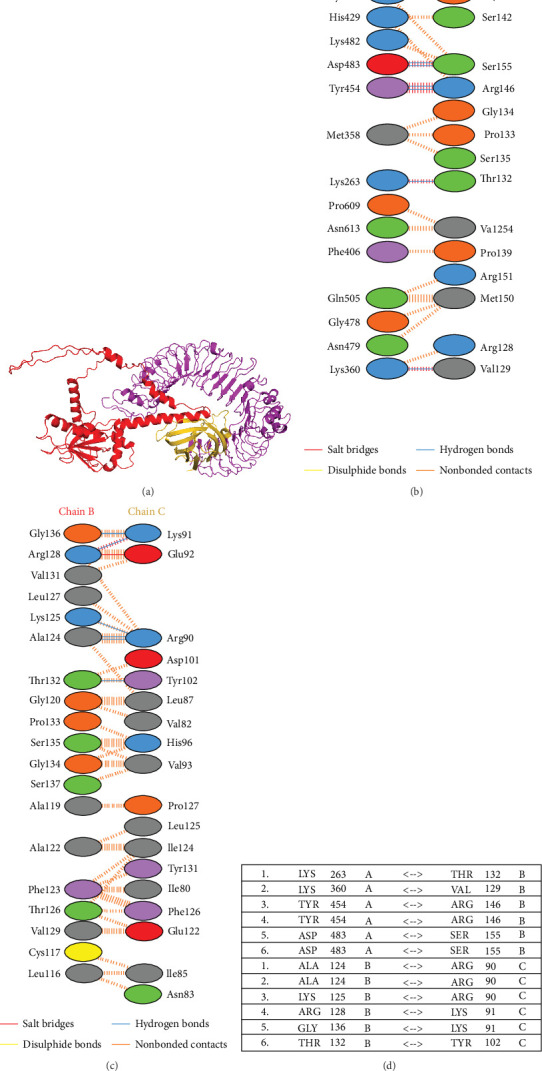
Representation of the molecular docking complex between chimeric multiepitope vaccine and Toll-like receptor 4 (TLR4) complex. (A) Three-dimensional visualization of the docking complex of the vaccine construct (in red) with *M. musculus* TLR4/MD-2/lipid IVa complex (in purpure). (B) Molecular interactions between chain A of the TLR4 receptor molecule and chain B of the vaccine construct and (C) interactions between chain B and chain C of the TLR4/MD-2/lipid IVa complex. (D) Hydrogen-bond interactions between chains A and B, and chains B and C.

Molecular docking studies have revealed that the interaction between the vaccine construct and the *M. musculus* TLR4/MD-2/lipid IVa complex is characterized by a binding energy of −16.3 kcal/mol, as calculated by the ClusPro web server (Figure 8A). This low binding energy suggests a strong affinity between the chimeric vaccine construct and the TLR4 receptor complex. The interface between the multiepitope vaccine (chain B) and TLR4 receptor (chain A) is defined by six hydrogen bonds and 61 nonbonding interactions. Additionally, when involving the multiepitope vaccine (chain B) and MD-2 of the TLR4/MD-2/lipid IVa complex (chain C), there is one salt bridge, six hydrogen bonds, and 86 nonbonding interactions, as shown in Figure 8B. Furthermore, PDBsum provided a graphical representation of the interacting residues between the multiepitope vaccine construct and TLR4 (Figure 8C) and a graphical representation of the interaction between the multiepitope vaccine and MD-2 (Figure 8D). These results suggest that the vaccine construct could interact with the TLR4/MD-2/lipid IVa complex of *M. musculus*. However, the same analysis was conducted with the human TLR4 complex, which yielded equally positive results, as depicted in (Figure S4).

### 3.9. Molecular Dynamics

**Figure 9:**
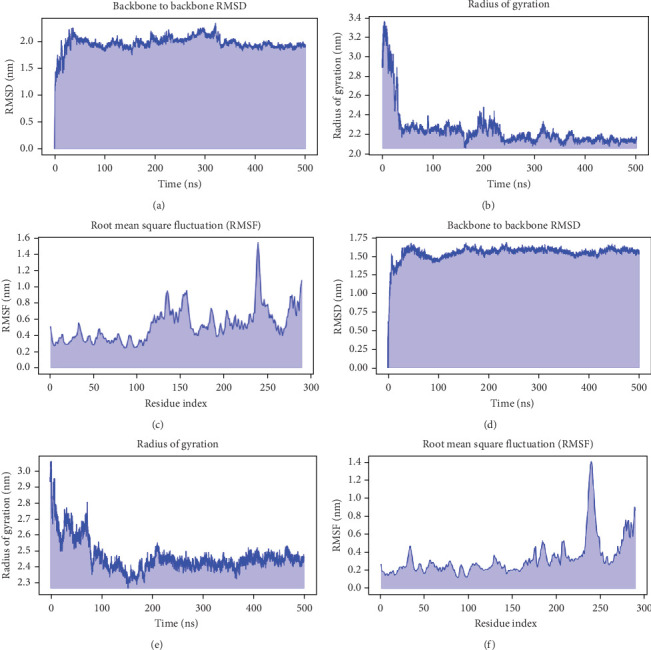
Assessment of the dynamic stability and residual flexibility of the modeled vaccine–Toll-like receptor 4 (TLR4) complex. (A) Root mean square deviation (RMSD) plot of the vaccine construct. (B) Radius of gyration (Rgyr) plot of the vaccine construct. (C) Root mean square fluctuation (RMSF) plot of the vaccine construct. (D) RMSD plot of the vaccine-TLR4 complex. (E) Rgyr plot of the vaccine-TLR4 complex. (F) RMSF plot of the vaccine–TLR4 complex.

The RMSD of atomic positions of the vaccine construct and the vaccine-TLR4 complex were analyzed based on the backbone atoms of the protein to measure the dynamic behavior and conformational stability from its initial to its final state. The average RMSD values were maintained at less than 2 nm throughout a 500 ns simulation, indicating stability after 10 ns for the free form and 25 ns when complexed with TLR4 (Figure 9A,D). This suggests the formation of a stable complex with minimal fluctuations. The Rgyr analysis also supports the development of a compact and stable structure of the vaccine construct and the complex (Figure 9). Additionally, a study of residual flexibility using RMSF for the C*α* atoms indicated reduced flexibility at the receptor-binding interface (Figure 9). Collectively, these results confirm that the construct, when associated with TLR4, achieves significant dynamic stability within the 500 ns of simulation timeframe.

### 3.10. Normal Mode Analysis

**Figure 10:**
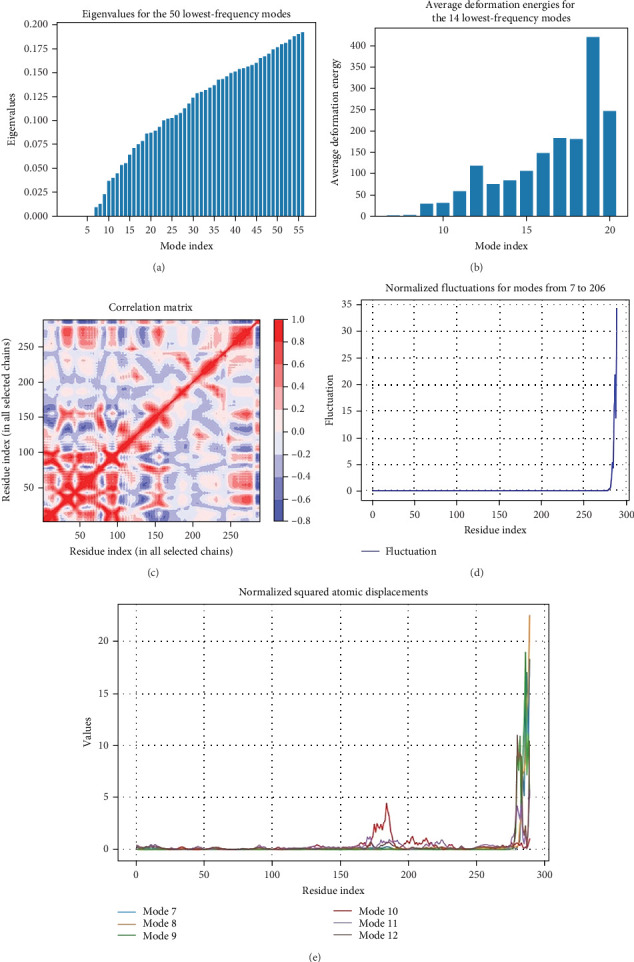
Normal mode analysis of the vaccine–Toll-like receptor 4 (TLR4) complex. (A) Eigenvalues for the 50 modes with the lowest frequencies. (B) Average deformation energies for the 14 modes with the lowest frequencies. (C) Covariance matrix illustrating the mobility correlations between residues pairs with the complex. (D) Fluctuation plot of the low-frequency modes. (E) Plot of atomic displacements associated with the low-frequency modes.

A normal mode analysis, facilitated by the WEBnma3 API program, was performed to determine the molecular stability and functional motions of the vaccine–TLR4 complex. A low eigenvalue of 8.5 × 10^−3^ indicated a high level of stability in the complex (Figure 10A). The deformation energies, which reflect the energy associated with each mode, were inversely related to the motion amplitude of that mode (Figure 10B). Molecular and atomic motions during the protein–protein interactions were examined using a covariance map that depicted correlations between each pair of residues in the vaccine–TLR complex (Figure 10C). Movements within different residue pairs were categorized as correlated (red), uncorrelated (white), and anticorrelated (blue). Fluctuations in atomic positions (Figure 10D) and atomic displacements (Figure 10E) of the six modes with the lowest frequencies were also identified. Deformability and fluctuations resulting from the simulation suggested that the vaccine-TLR4 complex maintains a balance of structural flexibility and stability.

### 3.11. Binding-Free Energy Calculation

The binding-free energy of the vaccine–TLR4 complex was determined using the MM-PBSA and MM-GBSA approaches. The total binding-free energy of the complex was calculated to be −275.87 kcal/mol using MM-PBSA. The gas energy phase had a negative value for the complex, whereas the solvation energy had positive values. Additionally, each component's energy contribution is detailed in Table 4.

### 3.12. Codon Optimization and In Silico Cloning of the Vaccine Construct

**Figure 11:**
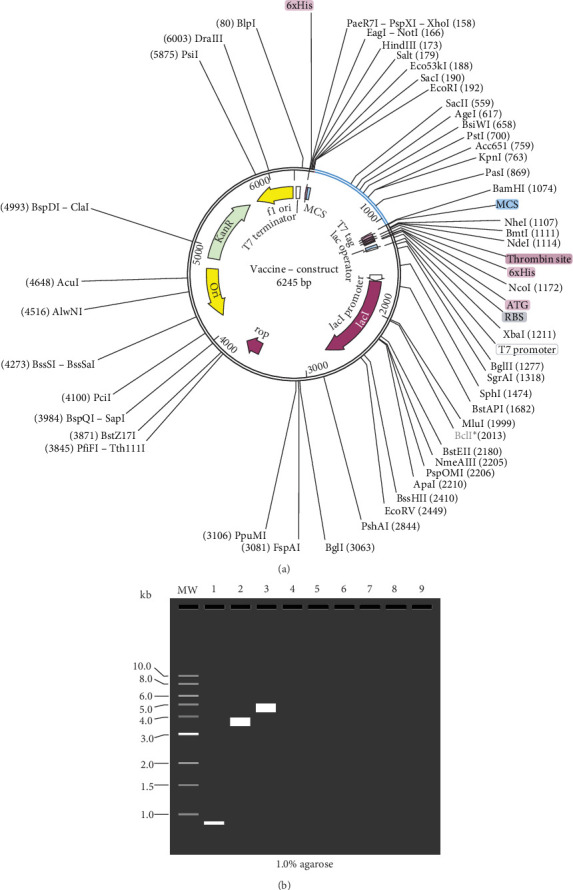
(A) In silico cloning of the vaccine construct sequence into the pET-28a (+) expression vector. The construct sequence (blue) was cloned between the vector's *EcoRI* and *BamHI* restriction sites (black). (B) 1% agarose gel electrophoresis simulation. The lanes show the molecular weight (MW), and the cloning results in positions 1–3, where lane 1, lane 2, and lane 3 represent the vaccine construct (888bp), the pET-28a (+) (5369bp), and the recombinant plasmid (6245bp), respectively.

The vaccine construct would be 888bp long, with a Codon Adaptation Index (CAI) of 0.86 and a GC content of 0,54%. Using SnapGene software version 7.2.1, the optimized construct was cloned into the pET-28a (+) expression vector between *EcoRI* (192) and *BamHI* (198). The total length of the pET-28a (+) with the inserted construct sequence was found to be 6245bp (Figure 11A). Additionally, the size of the cloning product was confirmed using the simulated agarose gel electrophoresis tool from SnapGene (Figure 11B).

## 4. Discussion

*Brucella* spp. are Gram-negative, intracellular pathogens causing brucellosis [100]. Among them, *B. canis*, one of the most prevalent species, is responsible for causing canine brucellosis. This condition is reported globally as a significant public health concern due to the intimate contact between dogs and humans [101]. Canine brucellosis is characterized by symptoms such as abortion, reproductive failure, and enlarged lymph nodes, and it may occasionally affect the osteoarticular system in dogs. In contrast, in humans, the disease manifests as a febrile syndrome, commonly known as Malta fever, with nonspecific symptoms such as splenomegaly, fatigue, and weakness [13]. There is currently no prophylactic for either canine or human brucellosis. Although some experimental vaccines for dogs have shown promise in terms of immunogenicity and protection, they rely on attenuated strains that could potentially be pathogenic and exacerbate transmission among canines and humans [102]. Innovative proteomic and bioinformatic methods enable the identification of virulence factors that play a role in the infectious process of these pathogens, offering potential targets for vaccine development [102]. The study of *B. canis* proteome is crucial as it provides an extensive protein analysis across various biochemical and pathological pathways, aiding the identification of novel drug targets. In this study, we employed reverse vaccinology approaches and subtractive genomics-based computational schemes to examine the complete proteome of *B. canis* strain RM6/66 for the construction of a new multiepitope vaccine candidate to evaluate its potential efficacy, including its physicochemical properties, safety, and immunogenicity.

The vaccine model was designed by overlapping MHC-I, MHC-II, and B-cell epitopes for mouse H2 alleles, integrated with specific connectors and adjuvant sequences for a chimeric vaccine as predicted by specialized and validated bioinformatic tool (Table S1). Overlapping epitopes were selected because their use makes it possible to induce both humoral and cellular immune responses [103], achieves higher population coverage [104], and results in a more compact construct [105–107] compared to the use of concatenated epitopes. These vaccine constructs are advantageous because they can contain more epitopes than those constructed with concatenated peptides [108], and these epitopes are conserved while avoiding repetition in the same sequence [107]. To prevent the loss of immunodominant epitopes, a threshold for the IC50 value was set during epitope prediction [108, 109]. MHC-I and MHC-II epitopes are crucial as they enable cytotoxic T cells to identify and eliminate infected cells, thus conferring broader immunity [110]. Meanwhile, humoral immune responses mediated by B cells involve antibody secretion to neutralize reactive pathogens and establish long-term immunological memory against future exposures [111]. T cells, both cytotoxic and helpers, activate cellular immune responses essential for halting the spread of disease, either by targeting infected cells for elimination or by secreting antimicrobial cytokines that contribute to sustained immunity for years or even decades [112]. Since the candidate must first be evaluated in a murine model, we used the mouse H-2 allele for epitope prediction. Mice remain a premier animal model for MHC research because their alleles are fully sequenced, well-characterized [113], and integrated into bioinformatic tools such as NetMHCpan. Although the dog leukocyte antigen (DLA) genomic regions are also completely sequenced with some alleles available for epitope prediction [114], detailed information regarding genomic differences among alleles remains limited [115]. Recent studies have analyzed the diversity of haplotypes [112, 116] and determined their association with diseases such as chronic enteropathy [117] and meningoencephalomyelitis [118]. As a result, one class I DLA allele is now available in NetMHCpan. Thus, the low knowledge of class I and II alleles restricts the prediction of epitopes for dogs. Among the six overlapping epitopes, two possess the ability to induce IFN-*γ*, indicating that IFN-*γ* enhances pathogen clearance when produced at elevated levels following vaccination [110, 119]. Furthermore, linkers are strategically incorporated to ensure that each epitope can trigger an independent immune response and to avoid the formation of novel epitopes that might disrupt the immune activity induced by the original epitope. Linkers also serve to enhance the stability and expression of the vaccine [120]. The EAAAK linker contributes rigidity, which supports the helical conformation of the construct, providing stability and functionality [66]. Conversely, the GSGSG linker offers flexibility due to the small size of its constituent amino acids, which promotes interaction between the protein domains [121]. Furthermore, adjuvants play a pivotal role in the vaccine formulation by enhancing the antigen-specific immune response, preserving peptide stability, and improving immunogenicity [122]. The CTB adjuvant, a nontoxic component of the CTB subunit, has an affinity for monosialotetrahexosylganglioside (GM1), a glycosphingolipid present in antigen-presenting cells such as macrophages, dendritic cells, and B cells. This ensures optimal engagement with the immune system. CTB is commonly employed to bind antigens, achieved either through genetic fusion or chemical conjugation, resulting in markedly augmented immune responses to the antigens [63, 64]. Conversely, TpD, an epitope known as a potent universal adjuvant to the CD4 T-cell helper, has been shown to efficiently activate human PBMCs in vitro and enhance immune responses in vivo in mice and nonhuman primates [123].

The inclusion of 3D structural data is critical for understanding biomolecular interactions between the proposed vaccine and the murine immune receptors. Tertiary structural prediction and validation of the vaccine's candidates were conducted using an array of computational tools. The vaccine construct showed promising attributes of quality and stability, as indicated by Ramachandran plot assessments and *Z*-score evaluations. Molecular docking assessed the binding affinity of the vaccine construct with the murine TLR4/MD2 complex, which was used instead of TLR4 only because MD2 is critical for stabilizing ligand binding to the TLR4 receptor, providing a more accurate representation of the physiological context for immune activation [124, 125]. The analysis revealed robust interactions with MD2 and significant vaccine-TLR4 binding, likely facilitated by hydrogen bonding and salt bridge formation that are conducive to a favorable immune response. Several studies support vaccine engagement with the TLR4/MD2 complex, which elicits a strong T-cell response and increases IFN-*γ* production [126–128]. Molecular dynamics and normal mode analyses were performed to investigate the vaccine-TLR4 complex's stability and dynamic behavior. Simulation results confirmed consistent binding stability, as indicated by RMSD, Rgyr, and RMSF metrics, underscoring the importance of stability for long-term immunity [129]. A normal mode analysis provided insights into eigenvalues, strain energy, displacements, fluctuations, and correlations between pairs of residues, demonstrating the vaccine construct's flexibility and stability in its interaction with TLR4/MD2 [130].

To date, the design of a multiepitope vaccine targeting *B. canis* has not been reported; however, similar efforts have been proposed against *B. melitensis* [131–133], *B. abortus* [35], and other *Brucella* species [134–137]. These studies employed molecular dynamics to validate the stability and subsequent immunogenicity of their vaccine constructs. In this context, the proposal to develop a multiepitope vaccine for *B. canis* is well-supported by bioinformatic research, as previously demonstrated for pathogens such as HCV, *P. penneri*, and *L. henipavirus* [138–140]. Considering the physicochemical properties and predicted immunogenic values, it can be inferred that this multiepitope chimeric vaccine is stable and shows promising results, indicating that it is likely to maintain its immunogenicity and stability during the purification and preparation stages. Furthermore, the predictions suggest that the vaccine is highly antigenic with minimal allergenicity and toxicity, making it a safe and robust candidate for inducing a strong immune response upon immunization. Interestingly, due to the monophyletic characteristics of the *Brucella* group, the immunogenicity induced by this candidate vaccine may provide cross-reactivity against several *Brucella* species, particularly *B. canis* strains (data not shown). However, these in silico results—based on H2 alleles for epitope prediction, which are fully sequenced and well-characterized—raise several questions. These include whether the predicted immunogenicity can be replicated in murine models and what adjustments would be required to transfer the vaccine's design from H2 alleles to canine leukocyte antigen (DLA) alleles, should it prove to be immunogenic.

In addition to the aforementioned characteristics, in silico immune simulation suggests that this vaccine can effectively stimulate both humoral and cellular immune responses (Figure S5). However, the limitations of this bioinformatic approach must be acknowledged, as reliance on in silico predictions may not fully capture the complexities of immune responses in living organisms. Genetic variability among individuals and potential differences in epitope presentation between species may also influence the vaccine's efficacy [141]. The next steps will involve conducting in vitro and in vivo tests to validate these findings and compare them with experimental data.

## 5. Conclusions

This study introduces a novel vaccine construct candidate with predicted immunogenicity against *B. canis*, designed to elicit a substantial immune response in the immunized host. Developing a vaccine to prevent canine brucellosis is essential, as it not only addresses the health of the canine population but also mitigates the risk of zoonotic transmission associated with this disease. These preliminary findings lay the groundwork for further validation through the expression and purification of the recombinant protein and its evaluation in in vivo experimental assays.

## Figures and Tables

**Table 1:** Characteristics of the potential proteins for developing of the multiepitope vaccine for *B. canis*.

Protein name	PSORTb results	Molecular weight (kDa)	Transmembrane helices	Topology	Antigenicity VaxiJen	Antigenicity ANTIGENpro
WP_004684587.1 MetQ/NlpA family ABC transporter substrate-binding protein	Cytoplasmic membrane	27.97	1	Globular + signal peptide	0.4340 Probable antigen	0.751838 Probable antigen
WP_004690079.1 Nitrate reductase subunit beta	Cytoplasmic membrane	58.14	0	Globular	0.5512 Probable antigen	0.824488 Probable antigen
WP_004692008.1 Bifunctional diguanylate cyclase/phosphodiesterase	Cytoplasmic membrane	78.88	0	Globular	0.4321 Probable antigen	0.099321 Probable not antigen
WP_006133077.1 Autotransporter adhesin BtaE	Outer membrane	75.53	0	Beta	0.7680 Probable antigen	0.883782 Probable antigen

**Table 2:** The properties of the multiepitope protein.

Tool/parameter	Value
Number of amino acids	289
Molecular weight (Daltons)	30.3 kDa
Theoretical pI	9.66
Instability index	30.21
Aliphatic index	84.15
Hydropathicity GRAVY	−0.117
SoLpro	0.52
Antigenicity (Vaxijen score)	0.74
Antigenicity (ANTIGENpro)	0.93
Allergenicity	Nonallergen
Toxicity	Nontoxic

**Table 3:** Discontinuous B-cell epitopes of vaccine construct.

No.	Residues	No. of residues	ElliPro score
1	A:K180, A:V181, A:E182, A:R183, A:D185, A:G186, A:T187, A:S188	8	0.935
2	A:V129, A:G130, A:V131, A:T132, A:P133, A:G134, A:S135, A:G136, A:S137, A:K138, A:P139, A:V140, A:I141, A:S142, A:A143, A:E145, A:R146	17	0.87
3	A:T118, A:A119, A:G120, A:S121, A:A122, A:F123, A:A124, A:K125, A:T126, A:L127, A:R128	11	0.82
4	A:G228, A:S229, A:G230, A:S231, A:T232, A:V233, A:S234, A:V235, A:G236, A:D237, A:K238, A:G239, A:A240, A:E241, A:R242, A:T243, A:I244, A:T245, A:N246, A:V247	20	0.754
5	A:A149, A:M150, A:Y153, A:G154, A:S155, A:G156, A:S157, A:Y158, A:V159, A:P160, A:P161, A:L162, A:S163, A:P164, A:L165, A:Q166, A:S167, A:A168, A:A169, A:Q170, A:A171, A:G172, A:K173, A:G174, A:S175, A:G176, A:S177, A:E178, A:R179, A:R189, A:T190, A:M191, A:R192, A:I193, A:I194	35	0.72
6	A:I271, A:G272, A:I273, A:P274, A:M275, A:G276, A:L277, A:P278, A:Q279, A:S280, A:I281, A:A282, A:L283, A:S284, A:S285, A:V287, A:A288, A:Q289	18	0.709
7	A:H14, A:N15, A:N90, A:N91, A:K92, A:T93, A:P94	7	0.627
8	A:K195, A:S196, A:S197, A:I198, A:P199, A:P200, A:V201, A:E202, A:G203, A:N204, A:E205, A:G206	12	0.621
9	A:A33, A:G34, A:K35, A:R36	4	0.56
10	A:V210, A:N211, A:V212, A:S213, A:K216	5	0.556
11	A:G55, A:S56, A:Q57, A:H58	4	0.507

**Table 4:** Estimated binding-free energy for the vaccine construct–TLR4 complex.

Energy contributions	Vaccine construct–TLR4 complex (kcal/mol)
Van de Waals energy	−402.54
Electrostatic energy	−2194.58
Gas phase energy	−2597.11
Solvation energy	2321.24

## Data Availability

Raw data will be available upon request to the corresponding author. Additional materials are available in the Supporting Information Files.

## Supporting Information

Supporting Information Table S1. The servers and databases used in the design of the multiepitope vaccine. Table S2. Cutoff values of the servers used in the design of the multiepitope vaccine. Table S3. Scores of the candidate's proteins in PSORTb, VFDB, and DEG. Table S4. List of vaccine residue pairs with the potential to form disulfide bonds, along with their *χ*^3^ angles and energy values. Figure S1. Intrinsic disorder of the vaccine construct predicted by IUPred3. The intrinsic disorder is evaluated based on the position of amino acids versus their score. Figure S2. Signal peptide prediction of the vaccine construct predicted by SignalP 6.0 server. Figure S3. Transmembrane topology prediction for the vaccine construct by DeepTMHMM server. Figure S4. Representation of the molecular docking complex between chimeric multiepitope vaccine and TLR4 complex. (A) Three-dimensional visualization of the docking complex of the vaccine construct (in red) with human TLR4/MD-2/lipid IVa complex (in purpura). (B) Molecular interactions between chain A of the TLR4 receptor molecule and chain B of the vaccine construct, and (C) interactions between chain B and chain C of the TLR4/MD-2/lipid IVa complex. (D) Hydrogen-bond interactions between chain B and chain C. Figure S5. Immune simulation with vaccine construct after two doses of vaccine. (A) Antigen and immunoglobulins after two doses. (B) B lymphocytes response. (C) CD4 T-helper lymphocytes count and (D) subdivided per entity-state (active, resting, anergic, and duplicating). (E) CD8 T-cytotoxic lymphocytes count and (F) subdivided per entity-state. (https://kraken.iac.rm.cnr.it/C-IMMSIM/).
