# Supplementary material for: In Silico Design and Characterization of a Multiepitope Vaccine Candidate Against Brucella canis Using a Reverse Vaccinology Approach
Source: J Immunol Res. 2025 Apr 15;2025:6348238. doi: 10.1155/jimr/6348238 (PMC12014272; doi:10.1155/jimr/6348238)
Supplement: Supporting Information — Table S1. The servers and databases used in the design of the multiepitope vaccine. Table S2. Cutoff values of the servers used in the design of the multiepitope vaccine. Table S3. Scores of the candidate's proteins in PSORTb, VFDB, and DEG. Table S4. List of vaccine residue pairs with the potential to form disulfide bonds, along with their χ3 angles and energy values. Figure S1. Intrinsic disorder of the vaccine construct predicted by IUPred3. The intrinsic disorder is evaluated based on the position of amino acids versus their score. Figure S2. Signal peptide prediction of the vaccine construct predicted by SignalP 6.0 server. Figure S3. Transmembrane topology prediction for the vaccine construct by DeepTMHMM server. Figure S4. Representation of the molecular docking complex between chimeric multiepitope vaccine and TLR4 complex. (A) Three-dimensional visualization of the docking complex of the vaccine construct (in red) with human TLR4/MD-2/lipid IVa complex (in purpura). (B) Molecular interactions between chain A of the TLR4 receptor molecule and chain B of the vaccine construct, and (C) interactions between chain B and chain C of the TLR4/MD-2/lipid IVa complex. (D) Hydrogen-bond interactions between chain B and chain C. Figure S5. Immune simulation with vaccine construct after two doses of vaccine. (A) Antigen and immunoglobulins after two doses. (B) B lymphocytes response. (C) CD4 T-helper lymphocytes count and (D) subdivided per entity-state (active, resting, anergic, and duplicating). (E) CD8 T-cytotoxic lymphocytes count and (F) subdivided per entity-state. (https://kraken.iac.rm.cnr.it/C-IMMSIM/). [file 6348238.f1.docx]

**Table S1:** The servers and databases used in the design of the multi-epitope vaccine.

| Server/Database | Function/Role | Available in |
| --- | --- | --- |
| NCBI GenBank database | Used to obtain the complete protein sequence of *Brucella canis*, specifically the reference RM66/6 strain [GCF_000740335.1]. | https://www.ncbi.nlm.nih.gov/genbank/ |
| PSORTb | Predicts the subcellular localization of bacterial proteins using amino acid sequences and a combination of analytical modules tailored for prokaryotic structures. It integrates sequence features, motif analysis, and machine learning to classify proteins into refined localization categories with high accuracy. | https://www.psort.org/psortb/index.html |
| Virulence Factors of Bacterial Pathogens Database (VFDB) | Analyze and compare the protein sequences with the stored virulence factors of pathogenic bacteria in the database to identify similarities or potential virulent features in the analyzed proteins. | http://www.mgc.ac.cn/VFs/main.htm |
| Database of Essential Genes (DEG) | Analyze and compare the protein sequences with the stored essential genes in the database to identify similarities. | http://origin.tubic.org/deg/public/index.php |
| VaxiJen v2.0 | Predicts protein antigenicity based on amino acid composition, using an alignment-independent approach to identify potential protective antigens. | https://www.ddg-pharmfac.net/vaxijen/VaxiJen/VaxiJen.html |
| TMHMM-2.0 | Predicts transmembrane domains by analyzing amino acid composition using a Hidden Markov Model. | https://services.healthtech.dtu.dk/services/TMHMM-2.0/ |
| ABCPred | Predicts B-cell epitopes in a protein sequence using artificial neural network. | http://crdd.osdd.net/raghava/abcpred/ |
| Immune Epitope Database (IEDB) analysis resource | Predicts T-cell epitopes (MHC I and MHC II) in protein sequences using diverse algorithms, with support for H-2 alleles. | https://www.iedb.org |
| ANTIGENpro | Predicts protein antigenicity by multiple representations of the primary sequence and machine learning algorithms | https://scratch.proteomics.ics.uci.edu |
| AllerTOP2.0 | Predicts the allergenicity of the protein construct, using a method based on auto cross covariance (ACC) transformation of protein sequences into uniform equal-length vectors. | https://www.ddg-pharmfac.net/AllerTOP/ |
| ToxinPred2 | Predicts the toxicity of protein constructs by comparing them against a primary dataset of known toxins. | https://webs.iiitd.edu.in/raghava/toxinpred2/ |
| IFNepitope | Predicts protein sequence epitopes capable of inducing IFN-gamma release from CD4+ T cells. It evaluates and ranks peptides or peptide libraries for their IFN-gamma induction potential through virtual screening. | http://crdd.osdd.net/raghava/ifnepitope/ |
| ProtParam | Provides information about various physicochemical properties of a protein construct, including its molecular weight, theoretical pI, amino acid composition, atomic composition, extinction coefficient, estimated half-life, instability index, aliphatic index, and GRAVY. | https://web.expasy.org/protparam/ |
| SOLpro | Predicts the solubility of a protein upon overexpression in E. coli using a two-stage SVM architecture based on multiple sequence representations. It outputs solubility predictions and associated probabilities. | https://scratch.proteomics.ics.uci.edu |

**(cont)**

| PSIPRED | Predicts the secondary structure of protein constructs, including helices, strands, and disordered regions. It uses advanced neural networks trained on protein sequence patterns to achieve high accuracy in structural predictions. | https://www.ebi.ac.uk/thornton-srv/databases/pdbsum/ |
| --- | --- | --- |
| IUPred3 | Predicts intrinsic disorder in proteins, identifying regions lacking a stable tertiary structure under native conditions. It integrates IUPred2 for disordered regions and ANCHOR2 for disordered binding regions, with additional functionality to detect regions affected by the redox state. | https://iupred3.elte.hu |
| AlphaFold2 | Predicts the three-dimensional structure of protein constructs using a deep learning model based on attention mechanisms. It utilizes a neural network architecture trained on large datasets of known protein structures to predict accurate protein conformations, significantly improving structural prediction accuracy and reliability. | https://alphafoldserver.com |
| GalaxyRefine | Refines predicted three-dimensional protein structures by performing repeated perturbations and overall structural relaxation using molecular dynamics simulations. | https://galaxy.seoklab.org/cgi-bin/submit.cgi?type=REFINE |
| ERRAT | Evaluates refined tertiary structures of protein constructs by verifying crystallography-determined structures. Error values are plotted using a sliding 9-residue window, based on non-bonded atom-atom interaction statistics in the structure. | https://www.doe-mbi.ucla.edu/errat/ |
| ProSA-web | Evaluates refined tertiary protein structures using the classic ProSA program’s algorithm. It analyzes quality scores within the context of all known protein structures, highlighting problematic areas using interactive 3D visualizations. | https://prosa.services.came.sbg.ac.at/prosa.php |
| MolProbity | Evaluates refined protein structures by assessing both the geometry and overall quality of the model. It uses a database of high-resolution structures to compare the refined structure against idealized conformations. The algorithm checks for steric clashes, bond angles, and distances, providing detailed feedback on problematic regions and suggesting improvements. | http://molprobity.biochem.duke.edu |
| PROCHECK tool | Evaluates refined protein structures by assessing their stereochemical quality. It uses a set of statistical criteria to validate bond lengths, bond angles, and overall geometry. The algorithm checks for common errors like irregular bond angles, improper geometry, and side-chain positioning, providing a detailed analysis of the protein’s structure quality | https://www.ebi.ac.uk/thornton-srv/software/PROCHECK/ |
| ElliPro | Predicts conformational B-cell epitopes by implementing three algorithms: (i) approximating the protein shape as an ellipsoid, (ii) calculating the residue protrusion index (PI), and (iii) clustering neighboring residues based on their PI values. | http://tools.iedb.org/ellipro/ |
| Disulfide by Design 2 | Introduces disulfide bonds between protein residues to enhance structural stability. The software predicts and analyzes the formation of disulfide bonds, focusing on improving thermal stability by targeting regions with favorable B-factor values. | http://cptweb.cpt.wayne.edu/DbD2/ |
| SignalP-6.0 | Predicts signal peptides using a neural network trained on protein sequences. It learns general protein features from context-based predictions without labels, aiding in specific property predictions like signal peptide identification. | https://services.healthtech.dtu.dk/services/SignalP-6.0/ |
| DeepTMHMM | Prediction of topology and classification of the protein construct using deep learning methods. | https://dtu.biolib.com/DeepTMHMM |

**(cont)**

| ClusPro 2.0 | A protein modeling tool that predicts the tertiary structure of protein-protein complexes from individual protein structures. It combines fast Fourier transform (FFT) docking with molecular dynamics simulations to predict optimal complex conformations, enhancing binding site prediction and complex stability for more accurate structural models. | https://cluspro.bu.edu/login.php |
| --- | --- | --- |
| RCSB Protein Data Bank | Used to obtain the TLR4/MD-2/lipid IVa complex from *Mus musculus*. | https://www.rcsb.org |
| WEBnma3 | A tool for predicting collective protein motions using normal mode analysis (NMA) and elastic network models (ENM). It allows comparative analysis of protein flexibilitye, validated against other methods like principal component analysis. | https://apps.cbu.uib.no/webnma3 |
| CHARMM-GUI | Configure, execute, and analyze molecular dynamics, energy minimization, system assembly, and free energy calculations of the interaction between TLR4 and the protein construct. | https://www.charmm-gui.org |
| EMBOSS Backtranseq | Converts protein sequences back into nucleotide sequences, aiding in codon optimization of vaccine constructs. | https://www.ebi.ac.uk/jdispatcher/st/emboss_backtranseq |
| GenScript Rare Codon Analysis Tool | Evaluates codon optimization by identifying rare codons in protein sequences to improve expression in specific organisms. | https://www.genscript.com/tools/rare-codon-analysis/ |
| Snapgene | *In silico* cloning of the vaccine construct and agarose gel electrophoresis simulation. | https://www.snapgene.com/ |
| Addgene | Used to obtain the pET-28a(a) vector. | https://www.addgene.org/ |
| C-IMMSIM | Simulation of the immune response of the vaccine construct in humans. | https://kraken.iac.rm.cnr.it/C-IMMSIM/ |

**Table S2:** Cut-off values of the servers used in the design of the multiepitope vaccine.

| Server | Cut-off values |
| --- | --- |
| Virulence Factors of Bacterial Pathogens Database (VFDB) | Identity > 35%  E value < 10-15 |
| Database of Essential Genes (DEG) | Identity > 35%  E value < 10-15 |
| NCBI-BLASTp | Identity < 25%  E value < 10-15 |
| VaxiJen v2.0 | Antigenicity > 0,4 |
| TMHMM-2.0 | TMHMM ≤ 2 |
| ABCpred | Score > 0,5 |
| IEDB MHC I and II | IC50 < 200nM |

**Table S3:** Scores of the candidate’s proteins in PSORTb, VFDB and DEG.

| Protein | PSORTb | | VFDB | | | DEG | | |
| --- | --- | --- | --- | --- | --- | --- | --- | --- |
| ID | Localization | Score | Virulence Factor | Identity | E-value | Essential gene ID | Identity | E-value |
| WP_004684587.1 | Cytoplasmic Membrane | 999 | IlpA | 44,79% | 1,20E-60 | DEG10020076 | 40,00% | 7,61E-63 |
| WP_004690079.1 | Cytoplasmic Membrane | 982 | narH | 55,01% | 1,00E-159 | DEG10110103 | 72,67% | 0 |
| WP_004692008.1 | Cytoplasmic Membrane | 788 | cdpA | 37,10% | 6,70E-74 | DEG10130392 | 35,37% | 7,42E-80 |
| WP_006133077.1 | Outer Membrane | 886 | btaE | 87,94% | 0,00E+00 | DEG10350172 | 50,00% | 1,13E-24 |

**Table S4:** List of vaccine residue pairs with the potential to form disulfide bonds, along with their χ3 angles and energy values.

| RESIDUE 1 | | RESIDUE 2 | | BOND | |
| --- | --- | --- | --- | --- | --- |
| Seq # | AA | Seq # | AA | χ3 | kcal/mol |
| 201 | VAL | 204 | ASN | 106,52 | 1,95 |
| 13 | TYR | 16 | THR | 112,6 | 1,97 |
| 58 | HIS | 62 | GLN | 115,96 | 2,21 |
| 211 | ASN | 214 | GLN | 96,06 | 2,43 |
| 3 | PRO | 8 | ASP | -83,86 | 2,7 |
| 88 | VAL | 95 | HIS | 84,96 | 3,24 |
| 84 | GLU | 103 | ALA | -103,00 | 3,46 |
| 87 | CYS | 98 | ALA | -108,78 | 3,8 |
| 15 | ASN | 91 | ASN | 125,32 | 3,9 |
| 87 | CYS | 99 | ALA | 90,62 | 4,21 |
| 85 | LYS | 101 | SER | 100,32 | 4,33 |
| 92 | LYS | 96 | ALA | -115,65 | 4,82 |
| 76 | ALA | 81 | ALA | -108,11 | 5,54 |
| 39 | ALA | 51 | VAL | 92,7 | 6,99 |


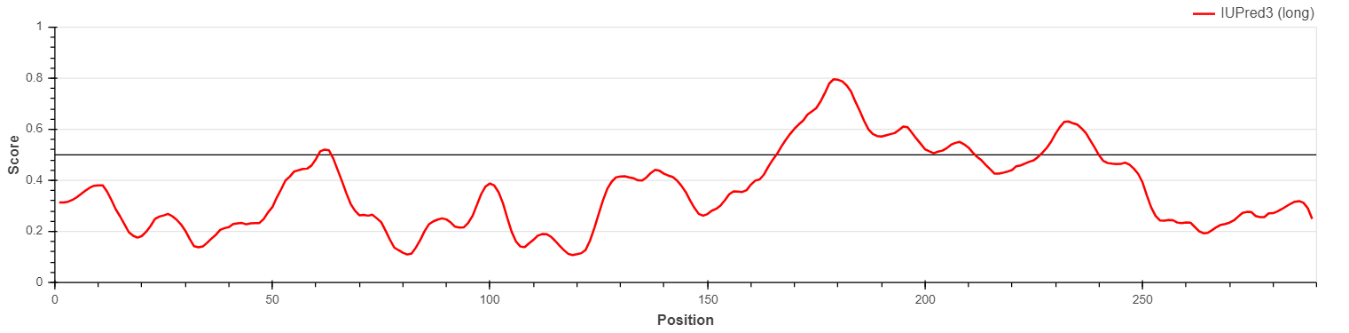


**Figure S1:** Intrinsic disorder of the vaccine construct predicted by IUPred3. The intrinsic disorder is evaluated based on the position of amino acids versus their score.


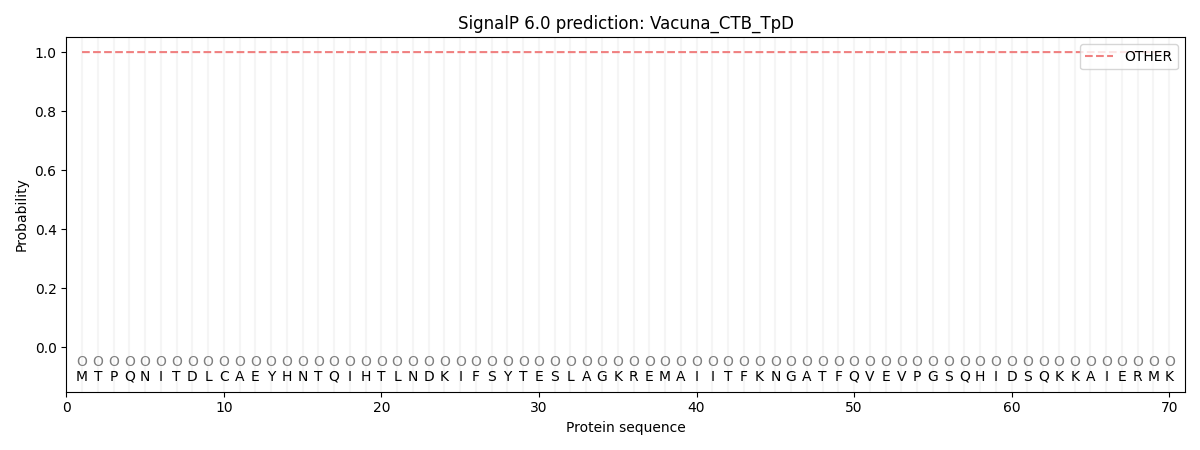


**Figure S2**: Signal peptide prediction of the vaccine construct predicted by SignalP 6.0 server.

**
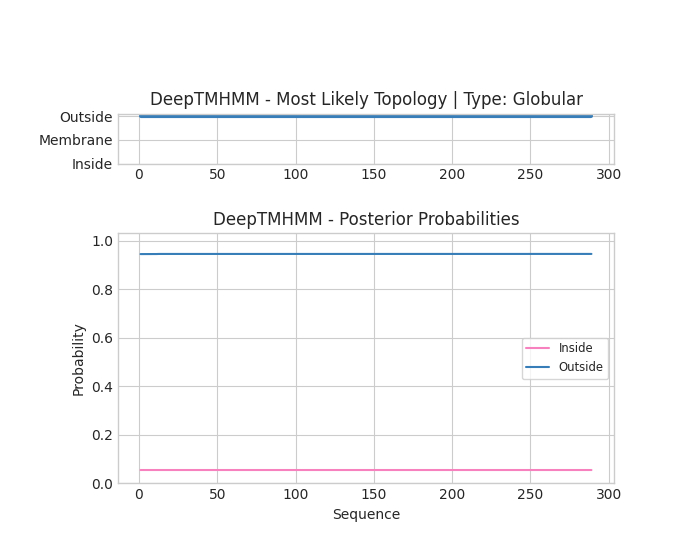
**

**Figure S3:** Transmembrane topology prediction for the vaccine construct by DeepTMHMM server.


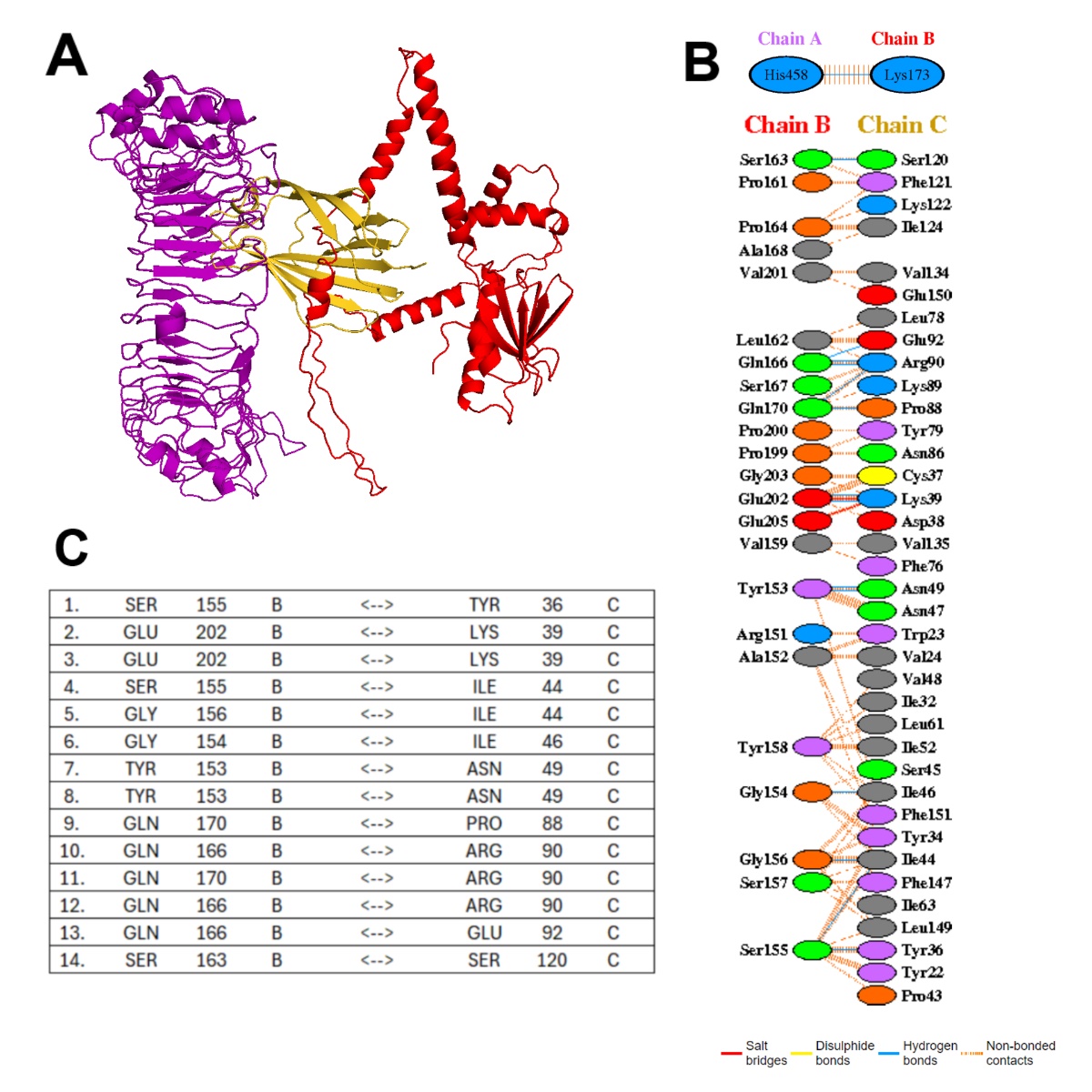


**Figure S4:** Representation of the molecular docking complex between chimeric multi-epitope vaccine and TLR4 complex. (A) Three-dimensional visualization of the docking complex of the vaccine construct (in red) with human TLR4/MD-2/lipid IVa complex (in purpure). (B) Molecular interactions between chain A of the TLR4 receptor molecule and chain B of the vaccine construct, and (C) interactions between chain B and chain C of the TLR4/MD-2/lipid IVa complex. (D) Hydrogen-bond interactions between chain B and chain C.


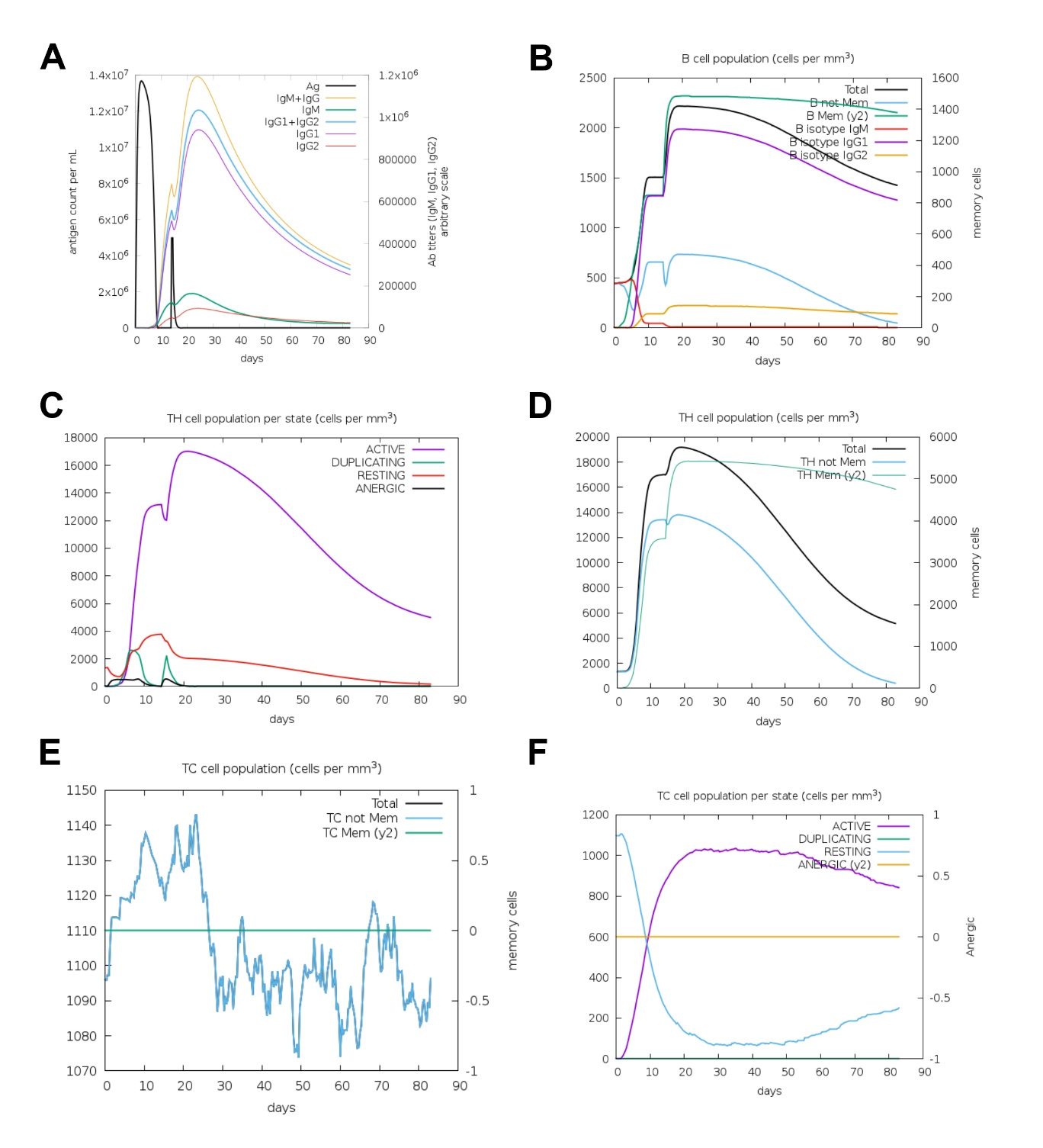


**Figure S5:** Immune simulation with vaccine construct after two doses of vaccine. (A) Antigen and immunoglobulins after two doses. (B) B lymphocytes response. (C) CD4 T-helper lymphocytes count and (D) sub-divided per entity-state (active, resting, anergic and duplicating). (E) CD8 T-cytotoxic lymphocytes count and (F) sub-divided per entity-state. (<https://kraken.iac.rm.cnr.it/C-IMMSIM/>)
